# Disrupting GPCR Complexes with Smart Drug-like Peptides

**DOI:** 10.3390/pharmaceutics14010161

**Published:** 2022-01-11

**Authors:** Maria Gallo, Sira Defaus, David Andreu

**Affiliations:** Department of Experimental and Health Sciences, Pompeu Fabra University, Barcelona Biomedical Research Park, 08003 Barcelona, Spain; maria.gallo@upf.edu

**Keywords:** peptide therapeutics, transmembrane peptides, GPCR oligomers, non-natural amino acids, cyclic peptides, retro-enantio

## Abstract

G protein-coupled receptors (GPCRs) are a superfamily of proteins classically described as monomeric transmembrane (TM) receptors. However, increasing evidence indicates that many GPCRs form higher-order assemblies made up of monomers pertaining to identical (homo) or to various (hetero) receptors. The formation and structure of these oligomers, their physiological role and possible therapeutic applications raise a variety of issues that are currently being actively explored. In this context, synthetic peptides derived from TM domains stand out as powerful tools that can be predictably targeted to disrupt GPCR oligomers, especially at the interface level, eventually impairing their action. However, despite such potential, TM-derived, GPCR-disrupting peptides often suffer from inadequate pharmacokinetic properties, such as low bioavailability, a short half-life or rapid clearance, which put into question their therapeutic relevance and promise. In this review, we provide a comprehensive overview of GPCR complexes, with an emphasis on current studies using GPCR-disrupting peptides mimicking TM domains involved in multimerization, and we also highlight recent strategies used to achieve drug-like versions of such TM peptide candidates for therapeutic application.

## 1. Introduction

G protein-coupled receptors (GPCRs) constitute the largest and most versatile superfamily of cell membrane-bound proteins, made up of seven trans-membrane α-helices (TM1 to TM7) [1,2,3] connected by intracellular (IL-1 to IL-3) and extracellular loops (EL-1 to EL-3), and coupled to an intracellular heterotrimeric G protein (e.g., Gs, Gi/o, Gq/11, G12/13) [4]. GPCRs are commonly grouped into six subfamilies (A-F) [5], based on sequence homology and functionality. Despite this apparent diversity, all GPCRs mediate their effects upon agonist-induced activation of the receptor at the extracellular site by a wide variety of ligands and then transduce the signal into intracellular responses [6]. Endogenous GPCR agonists are physically and chemically very diverse, including neurotransmitters (i.e., dopamine, serotonin), hormones (i.e., estrogen, angiotensin), proteins (i.e., chemokines), odors, photons, lipids (i.e., anandamide) or peptides (i.e., bradykinin), among many others [7]. Moreover, and more interestingly, ligand affinity for the GPCR primary (orthosteric) site and efficacy of activation can be increased or decreased by other effectors that bind to a separate (allosteric) site [8].

Given that GPCR signaling is involved in a diverse number of biological processes, GPCRs are considered ideal therapeutic targets [9] for a wide assortment of human diseases ranging from allergic rhinitis to pain, type-2 diabetes mellitus, obesity, depression, insomnia or cancer, to name just a few [10,11,12]; indeed, 34% of currently FDA-approved small-molecule drugs bind to GPCRs [13]. Originally described as cell-surface monomers that form a ternary complex with the extracellular ligand and the intracellular G protein [14], GPCR higher-order oligomers have in recent years been increasingly recognized as novel signaling units with functional properties distinct from their constituent receptors, thus opening up a new, only sparingly explored area of study within the GPCR field [15,16]. One possible strategy to probe into GPCR oligomerization and its impact on health conditions would consist in interfering in complex formation by means of exogenous synthetic peptides replicating TM domains involved in helix–helix interactions [17].

In this review, we consider the challenges and opportunities involved in disrupting GPCR oligomer formation by means of TM peptides targeting the complex interface, as a way to regulate oligomerization-dependent functions, and we also discuss strategies reported to improve the druggability of such peptide candidates in the context of cannabinoid-mediated pain management or CNS disorders.

## 2. GPCR Oligomers

The human genome encodes nearly 1000 different GPCRs, each one highly specific to a signaling pathway [18]. However, growing evidence indicates that many GPCRs can form active higher-order oligomers constituted by equal (homo) or different (hetero) monomers [19,20,21,22,23,24,25,26], with functional properties distinct from their protomer components [27] and generally involved in both healthy and pathological processes [28], thus making them ideal targets for the development and screening of novel drugs [29,30]. 

One of the first reported GPCR oligomers involved δ- and κ-opioid receptors that, when co-expressed, formed a stable heterodimer with properties not found in cells expressing the same receptor monomers [31]. Subsequently, many other GPCR homo- and/or hetero-complexes have been unveiled, often displaying unique characteristics. 

In many of these investigations the importance of TM helices in GPCR oligomerization has been demonstrated, portraying the GPCR complexes as dynamic species in which activation by the agonist induces a realignment of TM dimerization interfaces [32,33]. Indeed, it has been found that a dynamic equilibrium between monomeric and dimeric species can take place [34], modulated by ligand binding, which in turn can enhance or decrease heteromer interaction [35]. Therefore, while the minimal GPCR functional unit can be regarded as constituted by one monomeric receptor and one heterotrimeric G protein (1:1) [36], GPCR dimers can occur when: (i) two G proteins bind both dimer protomers (2:2) [37,38] or (ii) one G protein binds one protomer in the dimer (1:2) [39]. 

**Figure 1:**
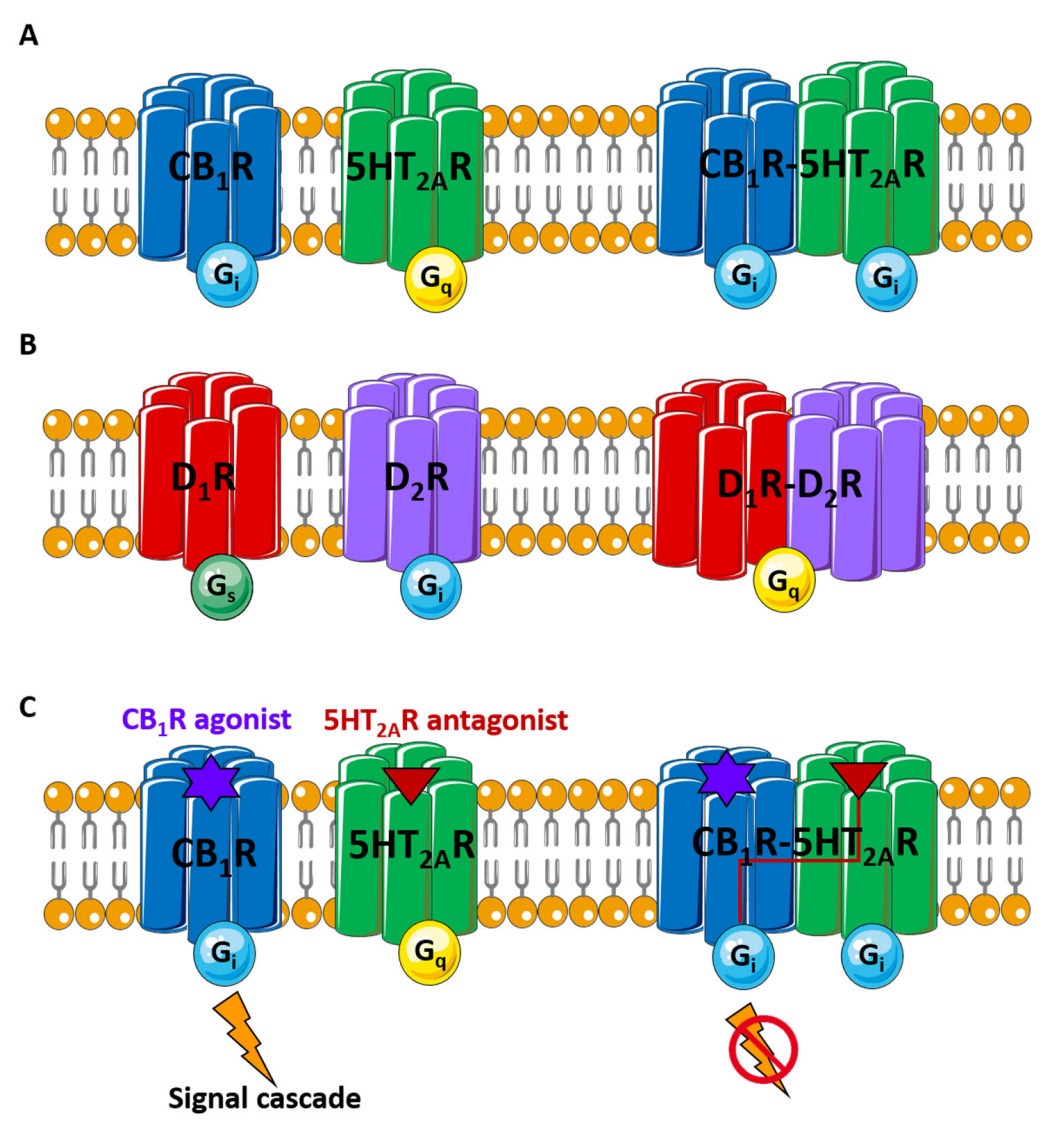
(**A**) The serotonin 5HT_2A_R and the cannabinoid CB_1_R monomers couple Gi and Gq proteins, respectively; when dimerized, however, 5HT_2A_R switches Gq protein with Gi; (**B**) The dopamine D_1_R and D_2_R monomers couple Gs or Gi, respectively; however, the heterodimer D_1_R-D_2_R couples Gq; (**C**) The serotonin 5HT_2A_R antagonist blocks the signal activation of the cannabinoid CB_1_R agonist when dimerized.

Another distinctive feature of some GPCRs is the switching of the G protein-coupled protomer when dimerization occurs. For instance, serotonin 5HT_2A_R couples Gq; however, heteromer formation by cannabinoid CB_1_R and 5HT_2A_R makes both receptors signal via Gi [40] (Figure 1A). In other words, some GPCR heteromers can couple G protein species different from those favoured by their protomers. Other reported examples are: (i) a heterodimer formed by dopamine D_1_ and D_2_ receptors that couples Gq instead of Gs or Gi [41] (Figure 1B); (ii) the heteromer formed by angiotensin AT_1_ and α_2c_-adrenergic receptors couples Gs instead of Gi or Gq [42]; and (iii) a melatonin MT_1_-MT_2_ receptor dimer that couples Gq instead of Gi [43]. 

Functionally, GPCR complexes can cause a positive or negative cooperation between promoters, i.e., ligand one binds to protomer one, enhancing or inhibiting, respectively, the affinity of ligand two for protomer two [44]. In general, intermolecular communication between GPCR homo- and heteromers tends to produce synergistic responses (i.e., functional cross-talk) [45]. A more singular phenomenon is cross-antagonism (Figure 1C), which occurs when a protomer antagonist blocks the signal activation of the other protomer [25,40,45]. Such a situation has been described for some GPCR complexes, including the metabotropic Gb_1_-Gb_2_ receptors [46], opioid δ-μ receptors [47], somatostatin SST_5_-dopamine D_2_ receptors [48], adenosine A_2A_-dopamine D_1_ receptors [49], orexin-corticotropin-releasing factor receptor [50] or angiotensin II AT_1_/dopamine D_2_ receptor [51].

Despite the extensive literature on GPCR oligomers, in most cases the assessment of their functionality has been only partially addressed and needs further investigation. In this context, chimeric peptide constructs have shown the ability to disrupt homo- and heteromer complexes, altering agonist-induced functionality and providing knowledge on the physiological role of GPCR receptor–receptor interactions [52,53,54,55].

## 3. Synthetic TM Peptides as Tools for GPCR Complex Exploration

The identification of protein–protein interaction interfaces constitutes a fundamental aspect in the study of GPCR complex formation [56], in that it can expand our understanding of the role that receptor oligomerization plays in intercellular communication or in some pathological conditions.

Increasing evidence indicates that specific TM helices are required for oligomerization, and that the synthetic peptides reproducing them are powerful tools to identify sequences essential for GPCR complexation and, by blocking their assembly, gain insights into the functional role of the complex [52,57,58].

For instance, Köfalvi et al. (2020) have recently studied how the adenosine-cannabinoid receptors, specifically the A_2A_R-CB_1_R heterotetramer interface, which also includes A_2A_R-A_2A_R and CB_1_R-CB_1_R homodimers, is established. To this end they have used computational modelling, with input from several biophysical and biochemical techniques, to design TM interference peptides reproducing each of the A_2A_R and CB_1_R TM1-7 helices. The synthetic versions, fused to the cell-penetrating HIV-Tat sequence, were tested by in vitro bimolecular fluorescence complementation (BiFC) experiments. Peptides replicating TM5 and TM6 of both receptors were able to disrupt the heterotetramer; thus, the involvement of their interfaces in the complex formation was confirmed. On the other hand, in the absence of the CB_1_R receptor, BiFC assays showed that the A_2A_R-A_2A_R homodimer was only disrupted by peptide A_2A_R TM6, while when A_2A_R was missing, CB_1_R TM4 was the only peptide disturbing CB_1_R-CB_1_R homodimer formation, altogether indicating that TM6 and TM4 sequences are involved in A_2A_R and CB_1_R homodimer interfaces, respectively [59]. 

Once the interfering peptides are identified, they can be used to investigate GPCR complex implications in numerous physiopathological disorders. As an example, Borroto-Escuela et al. (2018) found that rat A_2A_R TM5 peptide microinjection into the nucleus accumbens causes A_2A_R-D_2_R heteromer dissolution plus abrogation of the inhibitory effects of the A_2A_R agonist CGS21680 on cocaine self-administration, therefore confirming that the A_2A_R-D_2_R hetero-complex can be used as a novel target to treat cocaine disorders [53].

More examples where synthetic peptides replicating TM helices involved in dimerization have been shown to be able to split GPCR complex formations are included in Table 1. The in vitro (biophysical and/or biochemical) and in vivo assays used to confirm the existence of GPCR dimers in live cells and their implication (if known) in health disorders, are also presented.

### 3.1. TM Peptides: Challenges and Opportunities to Drug the Undruggable

Despite presenting great in vitro and in vivo potential in terms of efficacy, selectivity and safety, TM peptide disruptors of GPCR complexes are viewed as undesirable leads for therapeutic application due to their peptide-intrinsic poor pharmacokinetics, including low water solubility, high susceptibility to proteases, poor membrane permeability (including challenging physiological barriers such as the BBB), rapid clearance and immunogenicity [69]. These caveats notwithstanding, an array of peptide engineering strategies have been used over the years to improve druggability (see next paragraph and below) and can be also implemented in this case to develop optimized versions of GPCR complex-disrupting TM peptides and explore their therapeutic applications. 

**Figure 2:**
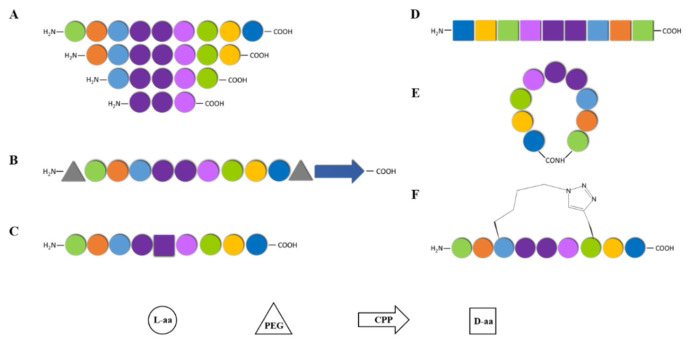
Useful synthetic strategies towards drug-like peptide design: (**A**) Peptide truncation at both N- and C- termini to identify the shortest active amino acid sequence; (**B**) peptide conjugation to PEG and/or CPP to enhance solubility and cell membrane permeation; (**C**) replacement of L- with D-amino acid to improve proteolytic stability; (**D**) retro-enantio approach to achieve a protease-resistant peptide with overall shape resemblance; (**E**) head-to-tail cyclization to increase half-life of peptides; (**F**) Stapling to constrain the peptide into a specific conformation.

The first useful item in the toolbox is trimming off some N-terminal or C-terminal residues until a minimally active primary structure can be established (Figure 2A). This reductionistic approach is particularly efficacious in the case of TM peptides since, by removing (almost invariably hydrophobic) residues from the cognate TM sequence, water solubility is improved [70,71,72], while synthesis time and costs are considerably reduced. Another strategy to improve peptide solubility is PEGylation, i.e., attaching several polyethylene glycol (PEG) units to the peptide lead structure (Figure 2B). PEG moieties, apart from being highly hydrophilic, are also good non-immunogenic spacers able to shield the peptide from proteolytic enzymes [73,74]. Additionally, related to this issue, the replacement of natural L- by non-natural D-amino acids is a common manoeuvre to improve peptide stability towards proteases in the digestive tract (e.g., trypsin), plasma and other biological fluids (Figure 2C). Furthermore, non-natural amino acids can enhance target affinity and selectivity by the induction or stabilization of secondary structure motifs (α-helices, β-sheets, β-turns) [75,76]. Along similar lines, the so-called *retro-enantio* approach can likewise give rise to peptides fully resistant to natural proteases. In a retro-enantio peptide both amino acid sequence and residue chirality are reversed relative to the parent structure, but despite these substantial changes the orientation of the side chains is preserved, hence a resemblance in overall shape (Figure 2D), while the inverted chirality curbs protease degradation, enhancing half-life and thus the potential as a new drug lead [77]. Moreover, the retro-enantio analogue tends to be less immunogenic than the cognate sequence [78]. Lastly, cyclization (head-to-tail (Figure 2E), side-chain-to-tail, side-chain-to-side-chain), including disulfide bond formation from native or (more frequently) engineered cysteine residues, is another quite valuable tool in the box that is regularly shown to reinforce serum stability [79]. Low membrane permeability, poor cellular uptake and inadequate homing specificity [80] are well-recognized snags that seriously jeopardize the success of peptide drugs. In this regard, a variety of drug carriers [81,82], with cell-penetrating peptides (CPPs) as a preeminent, successful example, have emerged as safe and efficient strategies to improve bioavailability. CPPs are a family of short peptide vectors with a remarkable inbuilt ability to traverse membranes, including important physiological hurdles such as the blood–brain or the skin barriers or the intestinal and nasal mucosae. Acting as drug delivery vectors (Figure 2B), CPPs can deliver into cells a plethora of payloads with therapeutic or diagnostic purposes, overcoming pharmacokinetic limitations and poor access to difficult areas, such as the central nervous system [80]. In sum, CPPs have become a powerful tool to address one of the main bottlenecks in drug development, namely the successful delivery of active compounds to target sites [80]. Moreover, in the specific case of GPCR complexes, CPPs can also determine the trajectory of the peptide disruptor into the membrane [80]. Peptide backbone modification is also an important and widely used approach to improve bioavailability [83,84]. Still another strategy to increase target affinity and cell uptake, as well as to protect against proteolytic degradation, involves *stapling*, whereby a synthetic brace (staple) introduced between two preestablished sites in the sequence helps to lock the peptide into a specific secondary structure (Figure 2F), thus reducing conformational entropy [85,86,87]. In the context of CB_1_R-5HT_2A_R complex disruption, the stapling approach has been recently applied [88], with preservation of in vitro GPCR oligomer disrupting activity along with improved proteolytic resistance.

Even though the resources in the peptide engineering toolbox just mentioned have allowed overcoming many of the factors limiting the therapeutic use of natural peptides with moderate success, for GPCR complex-disrupting peptides in particular, the goal of turning a canonical TM sequence into a viable drug lead may still entail a hazardous journey fraught with formidable obstacles. Even so, the latest literature reveals a slowly growing body of reports, including recent work from our group, where some of the aforementioned strategies are creatively applied to improve the druggability of GPCR complex-disrupting peptides. Two such accounts, dealing with the medical use of cannabinoids to fight pain and CNS disorder exploration and treatment, are discussed at some length below, illustrating the design and structural optimization process where TM peptides involved in GPCR dimer interfaces are developed into promising leads in two therapeutic areas with still unmet needs.

### 3.2. TM Peptides Restricting CB_1_R-5HT_2A_R Dimer for Cannabinoid Management in Pain Therapy

While cannabinoid-based therapy has proven effective in alleviating chronic pain [89,90,91], its psychotropic side effects such as memory loss, disorientation or dizziness are major obstacles in attempts to deploy cannabinoids as analgesics [92,93,94,95]. To this day, cannabinoids, both synthetic and naturally occurring, remain at the center of social, legal and medical debates concerning their therapeutic value, while the need for novel pain-alleviating medicines whose beneficial effects outweigh adverse ones is quite obvious in everyday clinical practice.

Various strategies have been investigated to minimize the unwanted outcomes of long-lasting cannabinoid exposure in patients undergoing cannabis-based therapies, so far to little avail. In line with this goal and with the topic of this review, we will now discuss work from our group and allied laboratories exploring the possibility of dissociating Δ^9^-tetrahydrocannabinol’s (THC) beneficial effects from its detrimental effects by using an optimized TM peptide disruptor to alter the CB_1_R-5HT_2A_R complex, i.e., the heterodimer formed by the cannabinoid CB_1_ and serotonin 5HT_2A_ receptors that is responsible for the undesirable cognitive impairment [40].

**Figure 3:**
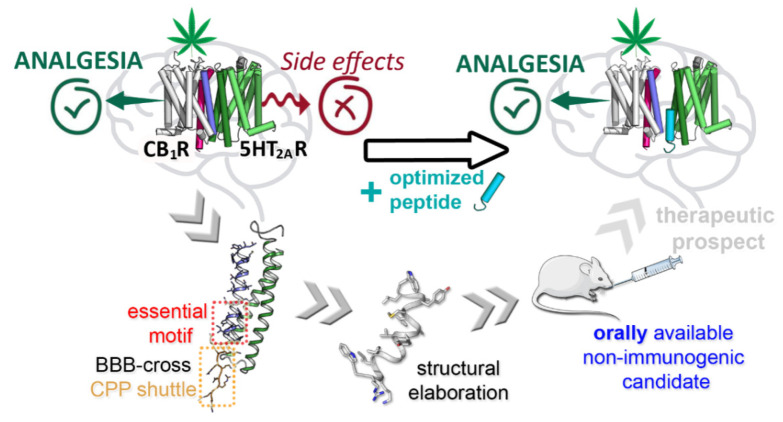
Development of a downsized, protease-resistant, orally available peptide compound, fused to an enhanced BBB-crossing CPP, which restricts the CB_1_R-5HT_2A_R heterodimer formation correlated to THC’s unwanted effects. (Reproduced with permission from [96]. American Chemical Society, 2021. Copyright © 2021 American Chemical Society, https://pubs.acs.org/doi/10.1021/acs.jmedchem.1c00484 (accessed on 27 December 2021). Further permissions related to the material excerpted should be directed to the American Chemical Society).

In order to identify the functional properties of the CB_1_R-5HT_2A_R heterodimer, we first used synthetic peptides reproducing the entire amino acid sequences of the CB_1_R TM5 and TM6 helices, fused to a cell-penetrating sequence derived from HIV-Tat, to disturb the formation of the heteromer [96]. These peptides were confirmed to be able to interfere with the CB_1_R-5HT_2A_R complex, both in vitro (BiFC, cAMP assay and p-ERK1/2 signalling) and in vivo (hot plate test and novel object recognition tests, after ICV administration in mice), allowing the selective activation of CB_1_R by THC [96]. However, their poor pharmacological profiles (i.e., long size, rapid proteolytic digestion, no trans-BBB permeability) were major drawbacks vis-á-vis any medical application. Therefore, with the goal in mind of preserving analgesic properties while minimizing cognitive side effects, our next step was to improve TM5 and TM6 peptide druggability by a combined effort aimed at reducing toxicity, prolonging serum half-life, avoiding immunogenicity, achieving BBB permeability and generally enhancing bioavailability, with oral activity as the final goal [96] (Figure 3).

After in silico identification of hotspots (i.e., close contact residues) in the CB_1_R-5HT_2A_R heterodimer interfaces, streamlined versions of the TM5 and TM6 peptides, i.e., containing mainly those amino acid residues predicted as involved in the interaction domain, were designed, synthesized and assayed, again juxtaposed to a CPP shuttle sequence (several size-optimized options tested). The results indicate that: (i) the downsized versions were as efficient as the original TM sequences in disrupting the CB_1_R-5HT_2A_R heteromer, and (ii) the previously used HIV-Tat CPP motif could be replaced, without loss in disruptive capacity, by a BBB shuttle peptide (BBBsP) sequence that ensured bioavailability of the novel analogs into their brain target [96].

In a last optimization round, candidates with reversed (i.e., non-natural D-residue) chirality were generated as the retro-enantio versions of the previous, shortened interfering peptides, again fused to a BBBsP motif also made up of D-residues. As mentioned earlier, by virtue of the retro-enantio approach the spatial orientation of the TM peptide side chains, hence the global shape, roughly resembles the canonical versions, and in tune with this, remains able to disturb the CB_1_R-5HT_2A_R heterodimer (in the BiFC assay). In addition, the BBBsP tag ensured efficient crossing of an in vitro BBB model and, given the all-D configuration, the peptide turned out to be highly resistant to serum proteases [96].

The last stage in our approach was evaluation in vivo: the two best-performing candidates were given intravenously to mice, co-administered with THC. While analgesic effects were observed for both peptides, only one of them was able to prevent THC-induced cognitive impairment. This peptide, wliymyayvaGilkrw (lower case one-letter notation for D-residues), was additionally shown to be orally available and non-immunogenic and is hence regarded as a highly promising lead in therapeutic approaches involving cannabinoid pain management without undesirable cognitive side effects [96].

### 3.3. TM Peptide Restricting A_2A_R-A_2A_R Dimer for CNS Disorders

Several functional G-protein heterodimers (Table 2, entries 1–7) and heterotrimers (Table 2, entries 8–10) with other GPCRs have been observed and described for the adenosine A_2A_ receptor (A_2A_R), playing significant roles in neurodegenerative diseases or drug abuse. Apart from heteromerization, A_2A_R homodimerization is well-recognized and raising attention as A_2A_R-A_2A_R homodimer inhibition has been recently reported to be involved in CNS disorders [97]. 

One strategy to explore A_2A_R-A_2A_R homodimer expression and its impact on brain disorders would consist, as in previous accounts above, in interfering in their formation by means of synthetic peptides replicating TM domains involved in helix–helix interactions. Thus, our laboratory recently ventured to assess A_2A_R- A_2A_R homodimer functionality using a peptide comprising the A_2A_R TM5 sequence, involved in the homodimer interface, fused to a linear HIV-Tat (47–57) CPP. Unfortunately, the outcome was unclear due to the fast in vivo degradation of the peptide.

**Figure 4:**
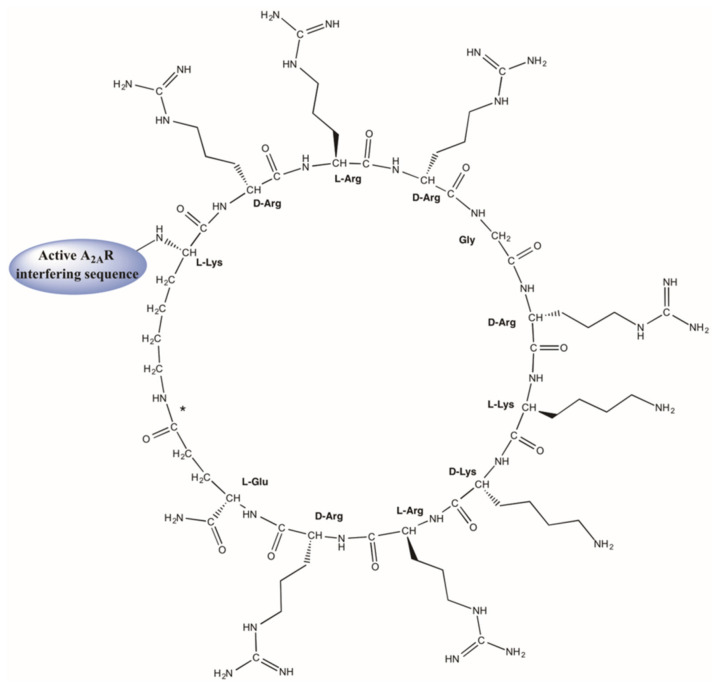
Design of an efficient A_2A_R-A_2A_R complex disruptor by combining a homodimerization-blocking sequence with a modified CCPP with improved pharmacokinetics properties (Figure adapted from [97]. MDPI, 2019). The Tat-like construct combines both L- and D-amino acids, and a side-chain-to-side-chain cyclization between the ε–amino group of the N-terminal Lys and the γ–carboxyl of C-terminal Glu (marked with an asterisk).

Different strategies to boost CPP protease resistance while preserving cell penetration efficiency have been developed [109]. One noteworthy approach is that afforded by cyclic versions of CPPs (CCPPs for short) such as cyclo Tat [110] or CPP12 [111]. These novel platforms have shown superior translocation ability over the linear versions and, importantly, a higher imperviousness to protease degradation. Based on such precedents, we set out to develop a construct where the TM5 peptide disruptor was linked to a cyclic Tat-like construct (Figure 4), with a view of ensuring an extended lifespan that might prove useful for future in vivo A_2A_R-A_2A_R homodimer studies. The hypothesis proved correct, and the CCPP afforded higher A_A2_R-A_2A_R disrupting capability than the linear HIV-Tat counterpart. Moreover, by including non-natural amino acids, the desired high proteolytic stability under physiological conditions was also achieved [97].

## 4. Conclusions

G protein-coupled receptors are preeminent among drug targets and, given the intensive research efforts devoted to them, are arguably likely to remain valuable sources of future pharmaceutical leads with important therapeutic indications. In this context, the emerging evidence of naturally existing GPCR oligomers has upended the conventional wisdom of the GPCR monomer as functional unit, opening up new horizons for pharmacological intervention. However, in contrast to well-studied GPCR monomer transduction, the mechanisms of GPCR oligomerization and, more importantly, their implication in health or disease, remain in most cases only partially elucidated and need to be studied further.

Several approaches have been made to gain insights into the functional relevance of GPCR oligomerization. For example, bivalent ligands that simultaneously bind the two physically interacting GPCRs orthosteric sites can be used as valuable pharmacological tools to study the quaternary structure of receptor dimers. Alternatively, mutant receptors that do not dimerize can be applied to probe the role of oligomers in the modulation of signal transduction. In this scenario, GPCR complex disruption by peptides reproducing TM sequences involved in dimer interfaces is increasingly recognized as a fruitful method for GPCR oligomer functional exploration, thus holding significant promise for the rational design of new GPCR-homing peptide drugs.

Despite their undisputed potential in medicinal applications, naturally occurring peptides have long been singled out as therapeutically problematic for intrinsic weaknesses such as low bioavailability and/or short in vivo half-life. To redress these shortcomings, an extensive array of structural modifications including C- or N-terminal truncation, use of non-natural amino acids, PEGylation or different cyclization tactics, to name just a few, have been successfully deployed over the years to fulfil the switch from natural (vulnerable) to engineered (drug-like), best-performing peptide leads. In the specific realm of TM peptides targeting GPCR oligomers, increasing research shows how, contrary to deeply rooted prejudice, educated structural elaboration of an initially naive sequence can enact its evolution into a full-fledged medicinal peptide entity. The first example of such transition shows that the analgesic properties of cannabinoids can be exploited while keeping their adverse side reactions (i.e., psychoactive effects and cognitive impairment) at bay by co-administration with a novel optimized CB_1_R-5HT_2A_R heterodimer-disrupting peptide. Such an approach has predictable potential in alleviating the plight of patients undergoing chronic pain medication. In this study, inclusion in the late-stage candidate of a substantially engineered CPP tag has turned out to be decisive, by endowing the peptide with BBB-crossing properties and hence access to brain cells where it can productively disrupt the CB_1_R-5HT_2A_R heterodimer. Similarly, an A_2A_R homodimer-disrupting construct, efficiently delivered by a protease-resistant cyclic CPP, was designed and produced for in vivo studies on the A_2A_R-A_2A_R homodimer implication in CNS disorders, including schizophrenia and Parkinson’s disease. Understanding its CNS delivery may pave the way to eventually harnessing some of the most challenging problems faced in the treatment of neurodegeneration. These encouraging developments are to be viewed as the first fruits in the quest for GPCR complex-disrupting agents, a field holding undoubtable promise for therapeutic application but where much work remains to be done.

## Figures and Tables

**Table 1:** GPCR complexes disrupted by synthetic TM peptides.

GPCR Complex	TMs Involved in Dimerization	Synthetic TM Disruptor Peptide	In Vitro/In Vivo Assays Performed	Patho-Physiological Implication	Ref.
A_2A_R-D_2_R	TM4/5 interface	A_2A_R TM5	BRET PLA Cocaine self-administration	Cocaine use	[53]
APJR-OX_1_R	TM4/5 interface	APJ TM4, TM5	BRET Co-IP	-	[60]
APJR homodimer	TM1, TM2, TM3, TM4	TM1, TM2, TM3, TM4	BRET FRET TIRFM Co-IP	-	[61]
A_2A_R-CB_1_R	TM 5/6 interface	CB_1_R TM5 TM6 A_2A_R TM5 TM6	BiFC BRET CODA-RET Glutamate release	Glutamate release	[59]
A_1_R-A_2A_R	TM 5/6 interface	A_2A_R TM4, TM5, TM6 A_1_R TM5 and TM6	BiFC PLA BRET cAMP production DMR	Neurodegeneration Neuroinflammation	[62]
CB_1_R-5HT_2A_R	TM 5/6 interface	CB_1_R TM5, TM6	BRET PLA BiFC NORT Hot plate test	Cognitive impairment	[40]
M_3_R homodimer	TM1, TM5, TM7	TM1-TM5-TM7	BRET	-	[63]
CCKR homodimer	TM6	TM6	BRET FRET	-	[64]
CCR5 homodimer	TM1, TM2, TM4	TM1, TM4	FRET Calcium determination	-	[65]
RhoR homodimer	TM1,TM2, TM4, TM5, H8	TM1, TM2, TM4, TM5	BRET cAMP production	Phototransduction	[66]
β_2_AR homodimer	TM1, TM5, TM6, H8	TM6	Adenylyl cyclase activity Densitometric analyses	-	[17]
SCTR	TM4	TM4	FRET BRET	Liver diseases	[55]
AT1aR-SCTR	TM1/2 interface TM4/4 interface	AT1aR TM1, TM4 SCTR TM2, TM4	BRET FRET cAMP	Hyperosmolality-induced drinking	[54]
FZD_6_ homodimer	TM4, TM5	TM4, TM5	FRAP FCCS	Cancer and neurologic disorders	[67]
MOR-DOR	MOR TM1	MOR TM1	Co-IP Immunoblotting Tail immersion	Morphine tolerance	[68]

Abbreviations: 5HT_2A_R, serotonin receptor type 2 A; A_1_R, adenosine receptor type 1; A_2A_R, adenosine receptor type 2A; APJR, apelin receptor; AT1aR, angiotensin receptor type 1a; BiFC, bimolecular fluorescence complementation; BRET, bioluminescence resonance energy transfer; cAMP, cyclic adenosine monophosphate; CB_1_R, cannabinoid receptor type 1; CCKR, cholecystokinin receptor; CCR5, chemokine receptor type 5; CODA-RET, complemented donor-acceptor resonance energy transfer; Co-IP, co-immunoprecipitation; D_2_R, dopamine receptor type 2; DMR, dynamic mass redistribution; DOR, δ-opioid receptor; FCCS, fluorescence cross-correlation spectroscopy; FRAP, fluorescence recovery after photobleaching; FRET, fluorescence resonance energy transfer; FZD_6_R, Frizzled-6 receptor; M_3_R, muscarinic acetylcholine receptor type 3; MOR, μ-opioid receptors; NORT, novel object recognition test; OX_1_R, orexin receptor type 1; PLA, proximity ligation assay; RhoR, rhodopsin receptor; SCTR, secretin receptor; TIRF, total internal reflection fluorescence; β_2_AR, adrenergic receptor type β_2_.

**Table 2:** A_2A_R complexes with other GPCRs and their implications.

Heteromer	Ligand	Implication	Ref.
A_1_R-A_2A_R	Caffein (A_1_R, A_2_R antagonist)	Drug tolerance	[98]
A_2A_R-D_2_R	A_2A_R antagonists, D_2_R agonists	Parkinson’s disease, schizophrenia, drug addiction	[99,100]
D_3_R-A_2A_R	CGS-21680 (A_2A_R agonist)	Schizophrenia	[101]
CB_1_R-A_2A_R	CBD (CB_1_R agonist)	Cognitive impairment	[102,103]
A_2A_R-mGlu_5_R	CHPG (mGluR5 agonist)	Parkinson’s disease	[23]
A_2A_R-H_3_R	RAMH (H_3_R agonist)	Autism, obsessive and compulsive disorder	[104]
A_2A_R-5HT_1A_R	CGS 21,680 (A_2A_R agonist), 8-OH-DPAT (5HT_1A_R agonist), SCH 58,216 (A_2A_R antagonist), methysergide (5HT_1A_R antagonist)	Dyskinesia	[105]
A_2A_R-D_2_R-mGlu_5_R	A_2A_R agonists, A_2A_R antagonists, D_2_R agonists, D_2_R antagonists, mGlu_5_R agonists	Psychosis, Parkinson’s disease, drug abuse	[106]
CB_1_R-A_2A_R-D_2_R	TBD	Endocannabinoid modulation	[107]
A_2A_R-D_2_R-NMDAR	α-synuclein	Neurodegeneration, neuroinflammation	[108]

Abbreviations: 5HT_1A_R, serotonin receptor type 1 A; 5HT_2A_R, serotonin receptor type 2 A; A_1_R, adenosine receptor type 1; 8-OH-DPAT, 8-Hydroxy-2-(di-n-propylamino)tetralin; A_2A_R, adenosine receptor type 2A; CBD, cannabidiol; CB_1_R, cannabinoid receptor type 1; CHPG, (R,S)-2-chloro-5-hydroxyphenylglycine; CG6-21680, 4-[2-[[6-amino-9(N-ethyl-beta-d-ribofuranuronaminoamidosyl)-9H-purin-2-yl]amino]ethyl]benzenepropanoic acid; D_2_R¸ dopamine receptor type 2; D_3_R, dopamine receptor type 3; mGlu_5_R, metabotropic glutamate receptor type 5; H_3_R, histamine receptor type 3; NMDAR; ‘N-metil-D-aspartate receptor; RAMH, (R)-(alpha)-(−)-methylhistamine dihydrobromide; SCH 58216, [5-amino-7-(2-phenylethyl)-2-(2-furyl)-pyrazolo [4,3-*e*]-1,2,4-triazolo[1.5-*c*]pyrimidine]; TBD, to be determined.
